# Outdoor stays—A basic human need except for older adults in residential care facilities? Researcher-practitioner interaction crosses zones and shows the way out

**DOI:** 10.3389/frdem.2024.1470691

**Published:** 2024-10-29

**Authors:** Susanna Nordin, Madeleine Liljegren, Martin Nilsson, Anna Bengtsson, Helle Wijk

**Affiliations:** ^1^School of Health and Welfare, Dalarna University, Falun, Sweden; ^2^Institution of Health and Care Sciences, University of Gothenburg, Department of Building Design, Architecture and Civil Engineering, Chalmers University of Technology, Gothenburg, Sweden; ^3^Health and Social Care Administration for the Elderly, City of Gothenburg, Gothenburg, Sweden; ^4^Department of People and Society, Swedish University of Agricultural Sciences, Alnarp, Sweden; ^5^Department of Quality Strategies Sahlgrenska University Hospital, Gothenburg, Sweden

**Keywords:** dementia, environmental evaluation tools, older adults, outdoor environment, outdoor stay, person-centered care and rehabilitation outdoors, researcher-practitioner interaction, residential care facility

## Abstract

The aim of this discussion paper is to show the way to the outdoors by shedding light on conditions in the physical environment enabling outdoor stays for older adults living in residential care facilities (RCFs). The origin was that outdoor stays is a basic human need and applies to everyone. However, despite extensive research on the health-promoting values of contact with the outdoors, it seems that for older adults in RCFs this is not met because they often have difficulty getting outdoors on their own. Therefore, the access to and the conditions of outdoor environments are discussed and exemplified through two cases based on evidence-based approaches, namely the principal model of four zones of contact with the outdoors, and the Swedish version of the Sheffield Care Environment Assessment Matrix (S-SCEAM). An interdisciplinary team, including both researchers and practitioners highlights future directions by showing the way to the outdoors on a national level with six suggested points. As a reader, you will gain increased knowledge about environmental qualities that support outdoor stays as well as initiatives that are needed to achieve equal conditions related to outdoor stays in RCFs.

## Introduction

This discussion paper stems from an identified need of improvement in access and design of outdoor environments for older adults living in residential care facilities (RCFs), so that the basic human need of outdoor stays can be met. We are an interdisciplinary group representing healthcare science, landscape architecture and environmental psychology, as well as practitioners planning RCFs, who have become aware of the challenges of outdoor stays for older adults with frail health. Despite recommendations to spend time outdoors for at least 2 h per week (White et al., 2019), this is seldom the case for older adults in frail health who tend to use the outdoor environment to a limited extent (The Swedish National Board of Health and Welfare, 2012). An identified contributing factor is the design of the physical environment. Therefore, the aim was to show the way to the outdoors by shedding light on conditions in the physical environment enabling outdoor stays for older adults living in RCFs, a group that often faces difficulties in getting outdoors on their own. As the Swedish National Board of Health and Welfare's national survey now also contains items about outdoor environments and activities along with routines for how the outdoor environment should be used, these issues are timely. The goal is that all older adults in RCFs should be offered outdoor stays on a daily basis all year round, therefore, this discussion paper can serve as support for municipalities and authorities in steering toward increased outdoor stays for older adults. In the present study, RCF refers to a special residence offering 24-h health and social care services for older adults in frail health. The care staff mainly consists of licensed practical nurses and nursing assistants, but registered nurses, physiotherapists, and occupational therapists can also be involved in providing care within RCFs (The Swedish National Board of Health Welfare, 2023). Two cases in urban contexts are used to illustrate supportive and hindering conditions related to outdoor stays based on existing evidence- and experienced based knowledge.

## Contact with the outdoors and health-related outcomes

Contact with the outdoors such as nature views (Sugiyama et al., 2022; Ulrich, 1984) and outdoor stays are basic human needs (Liljegren et al., 2024a) and applies to everyone, not least older adults in frail health who can benefit the most of having access to outdoor environments (Bengtsson and Lavesson, 2024; Ottosson and Grahn, 2006). One example is a multilevel cross-sectional study involving 290 older adults living in RCFs that showed significant associations between garden visits and self-perceived health (Dahlkvist et al., 2016). This is in accordance with the 2030 Agenda for Sustainable Development stating that accessible, safe, and inclusive green spaces can create opportunities for enriching the lives of the population in general, and older adults with disabilities in particular (United Nations, 2022). For instance, outdoor environments need to be supportive for persons with dementia, which is an essential part in creating dementia-friendly communities (Alzheimer's Society, 2023). A large body of research, including systematic reviews and intervention studies, has shown significant associations between nature and human physical, psychological and social health and wellbeing (Li et al., 2021; White et al., 2019; Yang et al., 2021; Zhang et al., 2017). For instance, outdoor environments can have a positive impact on mobility (Zieris et al., 2023), the immune system (Andersen et al., 2021), stress reduction (Litt et al., 2023), sleep quality (Shin et al., 2020), and reduce the risk of depression, loneliness, and isolation (Astell-Burt et al., 2022; Murroni et al., 2021). Having direct contact with nature also stimulates different senses (Bengtsson, 2015). Studies have also shown that the possibility to have a view from inside the building becomes a springboard that encourages persons to move outdoors (Musselwhite, 2018). Furthermore, spending time outdoors increases the level of physical activity compared to time spent inside and counteracts inactivity such as sedentary behavior and its negative consequences (Akpinar, 2016). Many of the residents spend most of their time indoors in the RCF (Rowles and Bernard, 2013), with a vast majority of waking hours spent in sedentary activities (de Souto Barreto et al., 2016; Parry et al., 2019). For older adults living in RCFs, access to outdoor environments is of particular importance since it can positively impact physical activity, orientation, social contacts, and overall wellbeing (Bengtsson et al., 2015; Brawley, 2001; Joseph, 2006). In dementia care, outdoor stays are linked to lower risk of falls (Detweiler et al., 2009), improved mood and sleep quality (Rappe and Kivelä, 2005), and increased social interactions (Raske, 2010). Moreover, garden activities can improve affective, behavioral, and cognitive factors among persons with dementia (Murroni et al., 2021; van der Velde-van Buuringen et al., 2021).

There is evidence that contact with nature also has positive effects for family members and healthcare staff (Ulrich et al., 1991). For instance, access to gardens have restorative benefits for staff working in healthcare settings (Liljegren et al., 2024a; Ulrich et al., 2008), increase their workplace satisfaction (Ulrich, 1999), and alleviate or prevent burnout among hospital staff (Cordoza et al., 2018; Mihandoust et al., 2021). Mihandoust et al. (2021) found that less exposure to nature views was related to higher burnout levels, especially in terms of emotional exhaustion and depersonalization. During the pandemic, outdoor stays was found to reduce stress and improve mental and physical health among staff (Gola et al., 2021; Iqbal and Abubakar, 2022; Loebach et al., 2022).

Older adults living in RCFs are characterized by variations in physical and cognitive health conditions (Schweighart et al., 2022). However, in several European countries there is an awareness of the increasing complexity in health conditions, with a growing need for high quality person-centered care and rehabilitation within RCFs (Barker et al., 2021; Spasova et al., 2018). Furthermore, older adults' opportunities to get out tend to decrease after moving into a RCF (Stoneham and Jones, 1997; The Swedish National Board of Health and Welfare, 2012), and outdoor stays has been reported by older adults themselves as the part they miss most after moving to a RCF (Liljegren et al., 2024a). Outdoor environments are of great importance and should be seen as a valuable resource. Unfortunately, there is a lack of incentives for outdoor stays as part of care and rehabilitation in RCFs, which is rooted in physical environmental obstacles as well as obstacles related to organizational challenges (Bengtsson, 2015; Liljegren et al., 2024a,b). Examples of physical obstacles are thresholds and heavy doors (Nordin et al., 2016), inadequate seating, and unsafe walkways (Anderzhon et al., 2007; Rodiek et al., 2014), absence of automatic doors and multi-story buildings (Liljegren et al., 2024b). Some organizational obstacles involve negative attitudes among healthcare staff toward spending time outdoors with the older adults, lack of mandate, and poor planning for outdoor stays as part of care and rehabilitation (Liljegren et al., 2024a).

## Theoretical and conceptual frameworks

**Figure 1:**
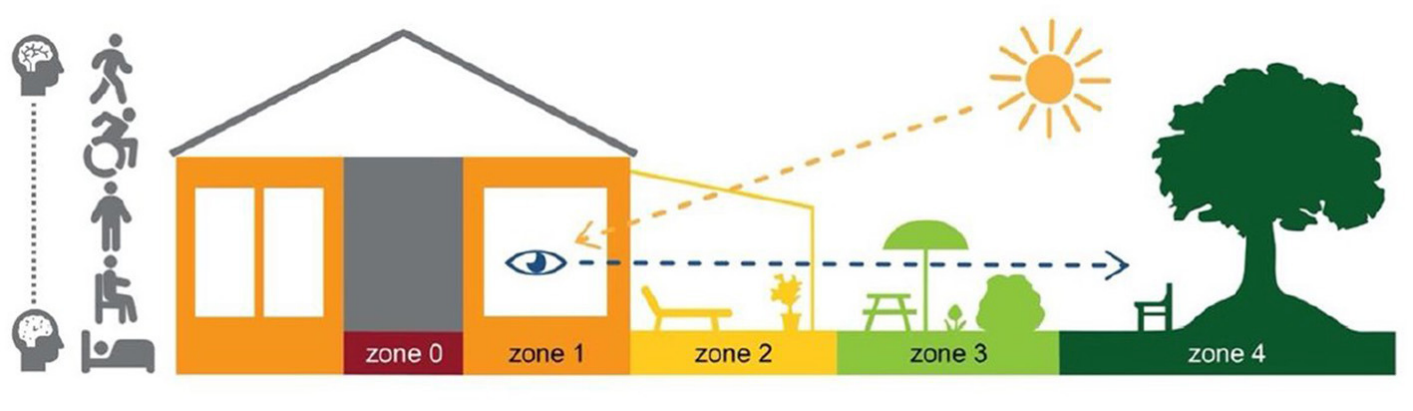
The zone model. Adaptation of original illustration by Anna Bengtsson.

Today, it is well recognized that care and rehabilitation for persons with frail health, including persons with dementia, should be based on a person-centered approach (McCormack et al., 2021; Zidén et al., 2024). Although the physical environment is essential to the opportunities for activities and social interactions, it is not always seen as an integral part of person-centered care and rehabilitation. However, there is an increasing awareness that person-centredness can be supported or hindered by environmental quality (McCormack and McCance, 2021), not least in RCFs (Calkins et al., 2022). To provide outdoor environments useful for older adults with varying physical and cognitive functional status, a conscious design of the environments is therefore required (Bengtsson, 2015; Chaudhury et al., 2018; Ng et al., 2023). For this purpose, a Swedish theoretical principal model of four zones of contact with the outdoors has been developed (Bengtsson, 2015) with each zone having a health-promoting value. The zones range from outdoor contact via windows (zone 1), in transitions between the indoor and outdoor environments (e.g. entrances, patios, balconies) (zone 2), in the garden (zone 3), to the surroundings (zone 4). The model also involves zone 0 which represents the indoor environment without contact with the outside world, i.e., without windows. Furthermore, older adults' opportunities for outdoor stays are related to their physical body positions such as lying, sitting, standing, and in motion with or without aids or personal support. Thus, the model includes a range of body positions. For this article, the model has been further developed to also include a range of cognitive status illustrated by a schematic drawing of a brain with different degrees of change in tissue. For example, a cognitive decline can influence a adults' possibility to express needs and take initiatives for outdoor stays or understand how to move between indoor and outdoor environments (Figure 1).

To ensure supporting aspects of the environments are considered, various assessment instruments can be used. Internationally, instruments that measure the qualities of physical environments in RCFs for older adults with frail health have been identified (Elf et al., 2017; Calkins et al., 2022). Of these, the majority focus on the indoor environment, although some instruments also include aspects on the outdoor environments such as the Quality Evaluation Tool (QET) including 19 design qualities for comfortable and inspiring design of outdoor environments in RCFs (Bengtsson, 2015), the Seniors' Outdoor Survey (SOS) (Rodiek et al., 2016), and the Swedish version of the Sheffield Care Environment Assessment Matrix (S-SCEAM) (Nordin et al., 2015). S-SCEAM have been translated and validated for Swedish RCFs and is based on the idea that a well-functioning RCF can improve the wellbeing of older adults and support them as frailty increases. The instrument assesses the physical environment from a person-centered perspective where the user's needs are represented by domains theorized as central in the occupancy of such environments: Cognitive support, Physical support, Safety, Normalness, Openness and integration, Privacy, Comfort, and Choice. It contains a large number of items, each of which describes environmental aspects that relate to different locations within RCFs, including gardens.

## Cases to illustrate quality evaluation by the application of tools

To illuminate varying conditions that support or hinder outdoor stays for older adults in RCFs, two cases from Sweden are presented with the use of the zone model and S-SCEAM. These two cases are chosen based on variations in terms of the prerequisites in the physical environments for contact with the outdoors, and they illustrate different environmental challenges that we have noted in relation to research and to practice, which will be described later. The zone model and S-SCEAM have not previously been used within the same study, however, the combination provided a comprehensive picture in this discussion paper. The RCFs are located in central Gothenburg, which is Sweden's second largest city with about 600,000 inhabitants.

### Case A

**Figure 2:**
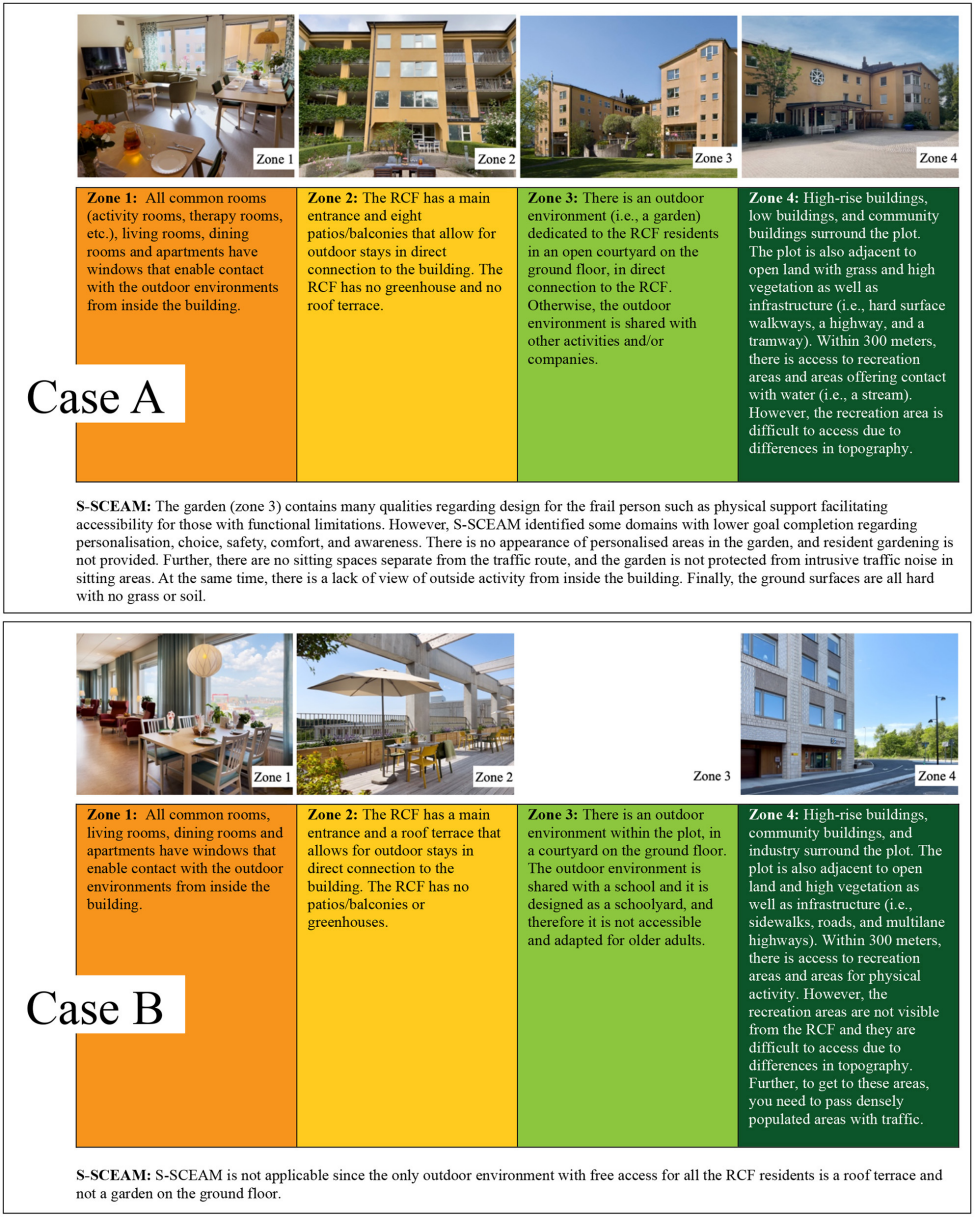
Description of Case A and Case B related to the zone model and S-SCEAM. Photo: Cordovan communication and P. Svensson, city of Gothenburg. Illustrated by M. Liljegren.

The RCF is located on a plot of 5,902 m^2^. The plot contains one building that is shared between the RCF and other type of group housing. The building has five floors with 40 apartments intended for older adults. See Figure 2 for description of case A related to the zone model and S-SCEAM.

### Case B

The RCF is located on a plot of 9571 m^2^. The plot contains one building that is shared between the RCF and a school. The building has seven floors with 100 apartments intended for older adults. See Figure 2 for description of case B related to the zone model and S-SCEAM.

The two cases presented in this discussion paper, showed interesting differences related to the varying conditions of the environments. These differences concerned both supportive and hindering aspects of the outdoor environments. In the following section, practical and theoretical aspects are discussed in relation to these two cases.

## Getting outdoors in practice

### Zone 1

Regarding zone 1, all common areas within the RCFs have outdoor contact via windows which is supported by recommendations stating that views of the outdoors from the interior should be maximized and distributed across multiple locations within the building (van den Berg et al., 2020). Although both cases have access to windows, there is a difference in what can be seen through the windows. In Case A, there are views toward greenery from all parts of the building, and the garden can be viewed from the indoor environment, whereas Case B have limited greenery views from the windows. Although the Case B have a panoramic view overlooking the harbor entrance and city center from some of the common rooms, the majority of the private rooms only have views toward the schoolyard and the facades of nearby buildings. Further, the roof terrace, i.e., the only outdoor environment with free access for all the RCF residents, cannot be seen through the windows. As previously mentioned, research has shown the importance of windows facing natural views. This aspect concerns from the older adults' private rooms and communal spaces. Previous research also shown that view of gardens and vegetation was a highly valued aspect when older adults themselves were asked what they preferred to look at through windows (Kearney and Winterbottom, 2006). Moreover, outdoor views from inside the building can be a springboard to get outdoors (Musselwhite, 2018). Considering that many older adults have frail health resulting in difficulties getting outside, access to zone 1 in terms of views through windows means a valuable contact with the outdoors, which therefore should be considered early in the process of building or renovation of RCFs.

### Zone 2

Case A have access to shared balconies for all older adults and visitors. Compared to private balconies, shared balconies are usually larger and provide better accessibility, but do not offer the same opportunity for integrity. In Case A, all balconies have thresholds for water drainage purposes, which is also the case for the roof terrace in Case B. However, thresholds are problematic as they pose obstacles for persons to reach the outdoors, not least for those using a wheelchair or a walker, something that has been highlighted in several studies (Bengtsson and Carlsson, 2006; Liljegren et al., 2024a,b; Nordin et al., 2016; Rodiek et al., 2014). In Case B, the only outdoor space with free access for all is a roof terrace, and internal signage is used to find its way there.

On the roof terrace, there is limited greenery, and a lack of weather and climate protection features. Overall, it is a challenge to choose suitable plants on roof terraces as they are exposed to different conditions such as sun, wind, rain and drought. For example, trees can provide natural shade, but these are difficult to establish in such outdoor environments. This was recently highlighted in an Australian study of urban apartment buildings showing that roof terraces had poor access to green space in terms of trees and vegetated landscapes (Bolleter et al., 2024). Regardless of which plants are chosen, roof terraces need to provide protection in various weather conditions (Chao et al., 2014; Cioffi et al., 2007; Heath and Gifford, 2001). Weather protection is essential to meet the needs of older adults in RCFs (Bengtsson and Carlsson, 2006). Moreover, since the roof terrace is situated high up in the building, it can pose challenges for older adults with both physical and cognitive disabilities to get there, and previous research has shown that proximity to outdoor spaces is important, especially for persons with dementia (van den Berg et al., 2020). Based on insights from these two cases, we argue that environments in zone 2 such as balconies and roof terraces require well-founded solutions and should not form the primary outdoor environment within RCFs. In other studies, it has been suggested that contact with the outdoors can be facilitated if there are several access points to reach the outdoors, instead of only one that may be located far from the older adults' apartments (Kearney and Winterbottom, 2006).

### Zone 3

In Case A, the S-SCEAM assessments identified environmental qualities in terms of physical support which is essential for accessing the outdoors for older adults with varying levels of functioning. The study by Potter et al. (2018) found that aspects related to both the physical environmental support, and the care staff influenced access to outdoor spaces for older adults in RCFs. For example, outdoor stays were restricted due to insufficient seating, uneven surfaces, or the need for assistance or permission from staff, which had a negative impact on their mood. Further, the S-SCEAM assessments showed that the garden was not protected from ambient environmental aspects such as traffic noise. This might be problematic for persons with cognitive disabilities as disturbing noise levels can be especially stressful (Joosse, 2012).

In case B, the ground floor outdoor environment was shared with children attending preschool and primary school. There may be benefits for older adults to share space with others such as cognitive stimulation and feelings of participation (Bengtsson, 2015), but it can also pose challenges as different groups have different needs. In addition, sufficient space is required both for school activities and for care and rehabilitation provided in RCFs. However, for schools in Sweden there are recommendations in terms of key figures of ~30–40 m^2^ for outdoor environment per child (The Swedish National Board of Housing, Building and Planning, 2024), something that does not exist for RCFs. Consequently, the school's outdoor environment is prioritized resulting in limited opportunities for outdoor stays for older adults' which can impinge on their choice and control. This is particularly relevant for case B, where the plot is divided between the RCF and a school. Since, at the time of construction, there were key figures for the school's outdoor environment but not for the RCF, it resulted in the outdoor environment at ground level being designed as a schoolyard and not in relation to the needs and preferences of the RCF tenants. In the city of Gothenburg, this problem has led to a local solution. A guideline with key figures for dedicated outdoor areas (within the plot) have recently been formulated for RCFs (Gothenburg City, 2024). However, the problem persists in other parts of Sweden, as well as in other European countries (Artmann et al., 2017).

### Zone 4

Regarding zone 4, there are level differences in terms of steep slopes in both cases. Case A has a staircase from the garden in zone 3 to the public recreation areas in zone 4, as well as sloping ground outside the entrance. In case B, there is a longer distance to recreation areas in zone 4, and it is not visible from the RCF. Further, the RCF is located in a dense area with traffic. Although older adults do not spend much of their time in public green areas beyond RCFs, they value access to these environments (Kearney and Winterbottom, 2006). For older adults with frail health, both sloping ground and long distances are obstacles making it difficult to reach public areas in zone 4. This have been reported in previous research as aspects that restricted the use of outdoor spaces, not least for those who are using wheelchairs or walkers (van den Berg et al., 2020), and even small grade changes seemed to limit outdoor stays (Kearney and Winterbottom, 2006).

## Theoretical perspectives on outdoor stays

Despite all the evidence of the importance of outdoor stays, it is still not considered a necessary activity at Swedish RCFs. Examples of hindering factors are staff being occupied with other tasks and have insufficient time to accompany the person, as well as their perception of safety risks in connection with outdoor stays (van den Berg et al., 2020). Consequently, systematic evaluations of outdoor access are given low priority. However, as researchers and practitioners, we believe that it is necessary to use evidence-based tools as part of ensuring and increasing the quality of outdoor environments in RCFs, which in turn can improve the living conditions of those living in such facilities. Furthermore, the importance of adopting a holistic and person-centered care approach is increasingly emphasized (Phelan et al., 2020). In this context, organizational factors such as leadership are essential for the care provided (Backman, 2018), which in turn will affect older adults' opportunities to access the outdoor environment.

With this paper, we want to highlight that the physical environment should be an integral part of care and rehabilitation, as environmental aspects have great potential to support or hinder the person-centered processes (McCormack et al., 2021). Because of this, and the fact that the construction and renovation of RCF involves large societal costs, we find it necessary to consider environmental qualities of the outdoor environment early in the planning process. The zone model provides a holistic perspective of contact with the outdoors where each of the four zones contributes to unique qualities. By using this model, environmental qualities can be related to the preferences, needs and prerequisites of older adults, and thus provide valuable knowledge about qualities that should be prioritized within the different zones (Bengtsson, 2015; Liljegren et al., 2024a,b). The S-SCEAM reflects core values of person-centredness such as opportunities for choice, comfort, integrity, and support for physical and cognitive frailty (Calkins et al., 2022; Nordin et al., 2015). The instrument can be used for detailed environmental assessments and identify specific aspects of the environment in need of improvement. For instance, environmental aspects that provide support for older adults with cognitive disabilities can be emphasized.

In sum, we argue that systematic evaluations are critical to the development of high-quality outdoor environments in RCFs, both the access to and the design of (see Table 1). The development can also lead to ground-level actions to change the working practices as part of overcoming the challenges of delivering outdoor care and rehabilitation. Today, there is already a shortage of healthcare staff in RCFs and the situation is expected to get worse. Hence, it is reasonable to assume that RCFs that invest in outdoor environments have a better chance of attracting new staff members.

**Table 1:** The use of systematic evaluations of physical outdoor environments in RCFs.

**Systematic evaluations can be used to:**
*Municipal level* •Create increased understanding of the target group's needs among, for example, decision makers, project managers, architects and landscape architects •Support interdisciplinary discussions between representatives of healthcare, landscape architecture, building planning and architecture •Guide the design of outdoor environments early in the commissioning process for RCFs •Assess the quality of existing RCFs and identify environmental characteristics in need of improvement before further planning •Assess the potential of existing buildings not previously used specifically as RCFs •Develop requirements when planning new buildings or renovating projects •Critically review drawings prior to planning new buildings or renovating projects
*Authority level* •Raise awareness of access to zones in RCFs •Guide the development of recommendations of access to each zone
*University level* •Map access to outdoor environments (by zone) to increase knowledge of the conditions for outdoor stays and outdoor work •Develop a working method for the design and planning of RCFs based on health-promoting environmental aspects •Set up a mobile research lab where an interdisciplinary group of researchers and practitioners visit different locations to study a certain phenomenon, such as the four zones of RCFs with the aim of increasing the knowledge of supporting environments

## Future directions

To show the way to the outdoors, we need to know where we come from. There is extensive and robust research on the health-promoting potential of the outdoors for older adults with frail health as well as for healthcare staff. Furthermore, there is research on supporting and hindering aspects in RCF environments, along with tools to support the planning of these environments. The importance of working from a person-centered approach has also previously been researched. The next step is therefore about the application of existing knowledge. See Table 2 for a presentation of conditions for how older adults can be offered outdoor stays on a daily basis.

**Table 2:** Take home messages for daily outdoor stay conditions.

**How older adults can be offered outdoor stays on a daily basis all year round**
1. National policy for outdoor stays: To support conscious leadership in RCFs, an overall policy for outdoor stays is required at a national level. In this way, equal access to outdoor environments throughout the country can be made possible 2. Person-centered care and rehabilitation outdoors: Incorporate outdoor work for the staff on a daily basis within the approach of person-centered care and rehabilitation. It is about performing the usual chores outdoors part of the time 3. Create good conditions for staff to work outdoors: Competence: Ensure that staff have sufficient competence to work outdoors. Routine: Ensure quality by developing a routine for outdoor stays in each municipality. Maintenance: Ensure attractive outdoor environments by daily arranging chairs and tables etc. and regular maintenance of vegetation 4. Access to all four zones: Key figures for outdoor spaces are required, and transitions between zones need to be ensured where all four zones must be accessible to older adults and staff, independent of geographic locations, all year round, and in all kinds of weather 5. Cooperation between authorities: To achieve equal and quality-assured outdoor environments and outdoor stays at a national level, there is a need for cooperation between national authorities such as an authority responsible for built environments, spatial planning and housing, and an authority responsible for high-quality health and social care for the population 6. Digital infrastructure: By developing a national digital infrastructure, data on access to outdoor environments can be related to health outcomes from already existing national quality registers used in RCFs

## Conclusion

In response to the opening title question: yes, outdoor stays is a basic human need even for older adults in RCFs, including persons with cognitive disabilities. This discussion paper highlights how older adults' needs of the outdoors in RCFs can be met by applying previous research on the health-promoting values of the outdoor environment in combination with six identified points. These points include the development of a national policy for outdoor stays, the promotion of person-centered care and rehabilitation outdoors as well as to create good conditions for healthcare staff to work outdoors. The points also include ensuring access to all four zones, cooperation between authorities, and the development of digital infrastructure.

The opportunities for outdoor stays for older adults in frail health is a matter of equality where such opportunities must be offered regardless of which municipality or RCF the older adult is living in. In this article, the researcher-practitioner interaction has crossed zones and showed the way to the outdoors. Now, municipalities and authorities need to join the discussion in Sweden and in other countries with similar challenges.

## Data Availability

The data analyzed in this study is subject to the following licenses/restrictions. The data is not available until later. Requests to access these datasets should be directed to: Susanna Nordin, snr@du.se.
